# *Listeria monocytogenes* associated kerato-conjunctivitis in four horses in Norway

**DOI:** 10.1186/s13028-015-0167-2

**Published:** 2015-11-09

**Authors:** Tobias Revold, Takele Abayneh, Hege Brun-Hansen, Signe L. Kleppe, Ernst-Otto Ropstad, Robert A. Hellings, Henning Sørum

**Affiliations:** Department of Companion Animal Clinical Sciences, Norwegian University of Life Sciences (previously Norwegian School of Veterinary Science), Ullevålsveien 72, Postbox 8146 Dep, 0033 Oslo, Norway; School of Veterinary Medicine, Addis Ababa University, Debre-Zeit, Ethiopia; Aktiv Dyreklinikk, 1484 Hakadal, Norway; Rikstotoklinikken Bjerke, Postboks 194, Økern, 0510 Oslo, Norway

**Keywords:** Equine, Cornea, Bacterial keratitis, Conjunctivitis, *Listeria monocytogenes*, Antimicrobial susceptibility

## Abstract

*Listeria monocytogenes* has been reported to cause various infectious diseases in both humans and animals. More rarely, ocular infections have been reported. To our knowledge, only two cases of *Listeria* keratitis have been described in horses. We report kerato-conjunctivitis in four Norwegian horses associated with *L. monocytogenes*. Clinically, all cases were presented with recurrent unilateral kerato-conjunctivitis. *L. monocytogenes* bacteria were isolated from swab samples from all cases, and cytology carried out in 3 cases was indicative of *L. monocytogenes* infection. The present report describes the first known cases in which *L. monocytogenes* has been isolated from keratitic lesions in horses in Norway. A potential risk factor may be feeding of silage or haylage, but other sources of infection cannot be ruled out. The phenotypic features including antimicrobial susceptibility and serotype of the isolates are described. Laboratory detection of *L. monocytogenes* demands extra caution since only low numbers of bacteria were detected in the eye-swabs, probably due to the low volume of sample material and the intracellular niche of the bacterium. A general poor response to treatment in all these cases indicates that clinicians should pay extra attention to intensity and duration of treatment if *L. monocytogenes* is identified in connection with equine kerato-conjunctivitis.

## Background

*Listeria monocytogenes* is an aerobic, Gram positive rod that can be found ubiquitously in nature, but is also capable of causing severe disease [1, 2]. Sheep, cattle, goats and chickens are considered to be the most susceptible species [3], but all animals including humans can be infected [1]. The bacterium is also recognized as an important foodborne pathogen [4]. Septicemia, encephalitis or abortions are the most common clinical manifestations of infection [2], but ocular symptoms may also be seen. In food animal species ocular symptoms are most commonly associated with infections affecting the central nervous system [5]. The term “silage eye” refers to listerial kerato-conjunctivitis, and may involve uveitis with hypopyon and miosis [6, 7]. In a questionnaire survey in England, “silage eye” was recognized as the most common clinical manifestation of listerial infection in dairy cattle, but the study did not include bacteriological data [6].

In humans, conjunctivitis is the most common ocular presentation of *L. monocytogenes* infection. Listerial conjunctivitis may either be endogenous in patients with meningitis, or exogenous [8]. Human cases of listerial keratitis and endophthalmitis have also been reported [8–12]. Human listerial endophthalmitis has been associated with a characteristic pigmented hypopyon and elevated intraocular pressure [8, 13].

Although listeriosis is less common in horses than in ruminants, there have been reports on abortions [2, 14, 15], intra-uterine infection [16, 17], meningo-encephalitis [18–20] and septicaemia [17, 18, 21–24]. The authors are only aware of two previously reported cases of equine kerato-conjunctivitis associated with *L. monocytogenes* [25, 26]. This report describes four cases of equine kerato-conjunctivis in Norway. The clinical course of the disease, as well as microbiological features and antimicrobial susceptibility (both in vitro *and* in vivo) of *L. monocytogenes* isolated from all 4 cases are described.

## Case presentations

### Case 1

A 12-year-old brown Standardbred mare with a 12 months history of unilateral recurrent kerato-conjunctivitis was presented to the field practice of the Norwegian School of Veterinary Science (NSVS). The horse was fed plastic wrapped silage and a commercial concentrate mix. Two previous attempts to treat the horse with topical fucidic acid eyedrops (Fucithalmic^1^) and oxytetracyclin/polymyxin B (Terramycin-Polymyxin B^2^) eye ointment were unsuccessful.

The ophthalmic examination was performed using a focal light source and revealed mild blepharospasm of the right eye with a mild mucopurulent discharge. The palpebral conjunctivae were moderately hyperaemic and chemotic. Direct and consensual pupillary light reflexes were normal, and no sign of miosis or aqueous flare were seen. Multiple grayish-white sub-epithelial opacities were observed in the central part of the cornea, with an irregular overlying epithelial surface in an area of approximately 5 × 5 mm. This area was surrounded by a diffuse stromal edema covering approximately half of the cornea. A bacterial swab was collected from the corneal lesion, and transferred to a sterile transport medium. The fluorescein dye test was negative.

The preliminary diagnosis was bacterial kerato-conjunctivitis, and treatment with chloramphenicol eye ointment (Chloramphenicol^3^) 3–4 times daily was initiated. *L. monocytogenes* and *Micrococcus* sp. were cultured from the bacterial swab. The *L. monocytogenes* was considered to be of potential clinical relevance, and was found to be sensitive to chloramphenicol. However, no improvement was observed upon re-examination 15 days later. A new bacterial swab confirmed the continuous presence of *L. monocytogenes*. Treatment was altered to topical ampicillin (Pentrexyl 500 mg^4^ in 5 ml sterile water), gentamicin eyedrops (Garamycin^5^) and diclofenac eye drops (Voltaren Ophtha^6^); all four times daily. In addition, the horse was treated with systemic procaine penicillin (Penovet vet^7^; 20 000 IU i.m.) twice daily and gentamicin (Genta-Kél^8^; 6.6 mg/kg i.v.) once daily; both for 5 days. A continuous gradual improvement was seen until the horse was deemed sound by the owner around day 55, and all treatment was discontinued.

At day 69, however, symptoms similar to those described at day 1 recurred, and again, *L. monocytogenes* and *Micrococcus* sp. were cultured. Treatment with topical ciprofloxacin eyedrops (Cilox^9^) and diclofenac eye drops [Voltaren Ophtha (see footnote 6)]; both four times daily, was initiated. At day 114 the horse was hospitalized at the NSVS equine clinic.

Ophthalmic examination at the NSVS confirmed abnormal findings restricted to the cornea and the conjunctiva. The fluorescein dye test was still negative. Bacterial swabs were collected both prior to and after epithelial scrapings for cytology, which resulted in considerable epithelial detachment. *L. monocytogenes* was cultured from both bacterial swabs, but the highest numbers were found prior to scraping. No fungi were cultured. Cytology revealed a high number of rods associated with the squamous epithelial cells that were seen both as aggregates and isolated. No neutrophils or fungi were seen.

A sub-palpebral lavage system attached to a continuous infusion pump system^10^ was inserted through the upper eyelid. Benzylpenicillin sodium (Penicillin 600 mg^11^ in 5 ml sterile water) and gentamicin [Garamycin (see footnote 5)] eye drops were mixed daily and used in the infusion pump. In addition, the horse was given systemic procaine penicillin [Penovet vet (see footnote 7); 20,000 IU i.m.] twice daily for 10 days, and systemic vedaprofen [Quadrisol vet (see footnote 2); 1 mg/kg after an initial dose of 2 mg/kg] once daily for 15 days.

At day 118, the footplate of the lavage system was noted to have caused a superficial ulceration in the dorsal cornea. The infusion set was therefore removed, and a new set was inserted in the nasal part of the lower eye lid, thereby protected by the membrana nictitans. The corneal lesion created by the lavage system healed uneventfully within a week, indicating a normal corneal healing ability.

At day 128, at which no bacteria were identified in a follow-up cytological evaluation, the apparently irritating topical benzylpenicillin sodium treatment was discontinued. Topical treatment with gentamicin [Garamycin (see footnote 5)] eye drops via the lavage system was maintained. The following days the inflammation was gradually reduced. At day 140, severe kerato-conjunctivitis recurred. Slides for cytological examination were prepared, of which one contained a high number of bacteria morphologically identical to *L.monocytogenes* associated with squamous epithelial cells. Interestingly, such bacteria were not seen on three further slides. The combined treatment with benzylpenicillin sodium [Penicillin 600 mg (see footnote 11) in 5 ml sterile water] and gentamicin [Garamycin (see footnote 5)] eye drops via continuous flow was restarted, but financial constraints made further treatment at the clinic impossible. Due to the nervous disposition of the horse, enucleation was not considered to be an option.

The horse was therefore released from the clinic against medical advice on day 150, and treated by the owner with oxytetracyclin/polymyxin B [Terramycin-Polymyxin B (see footnote 2)] eye ointment three times daily in an attempt to control the infection. Four weeks later, the horse was involved in a car accident and had to be euthanized due to its injuries.

### Case 2

A 7-year-old grey Connemara gelding with a history of recurrent unilateral kerato-conjunctivitis the last 6 months was referred to the NSVS. The pony was fed hay and a commercial concentrate mix, but was also fed plastic wrapped haylage from the ground of the paddocks at daytime. Although treatment with chloramphenicol eye ointment [Chloramphenicol (see footnote 3)] and diclofenac eye drops [Voltaren Ophtha (see footnote 6)] appeared to be effective, clinical signs recurred repeatedly after treatment withdrawal. The pony exhibited a marked blepharospasm of the left eye, a moderate periorbital edema with moderately hyperemic and chemotic palpebral conjunctivae, combined with a mild serous discharge. Slightly nasal to the centre of the cornea, there was a faint, diffusely outlined greyish-white epithelial and sub-epithelial opacity of approximately 2–3 mm diameter. There were no signs of uveitis. A bacterial swab was collected prior to the fluorescein dye test, which revealed a strong dye uptake in the centre of the lesion. There were no signs of neovascularisation. A corneal scraping for cytological evaluation resulted in epithelial detachment in an area of approximately 4 × 4 mm. The preliminary diagnosis was kerato-conjunctivitis of possible viral aetiology. Treatment with idoxuridine eye ointment (Iducher^12^) four times daily, fucidic acid eye drops [Fucithalmic (see footnote 1)] twice daily and diclofenac eye drops [Voltaren Ophtha (see footnote 6)] three times daily was initiated. The cytological findings indicated possible listeria keratitis. This was confirmed by culturing of *L. monocytogenes*. Based on the results of the sensitivity testing, topical antibiotics were changed to oxytetracyclin/polymyxin B [Terramycin-Polymyxin B (see footnote 2] three times daily, and antiviral and anti-inflammatory treatment was continued.

On day 12, the opacities were hardly visible, and there was no discharge or signs of conjunctivitis. Treatment with oxytetracyclin/polymyxin B [Terramycin-Polymyxin B (see footnote 2)] and idoxuridine eye ointment [Iducher (see footnote 12)] was continued for another 7 days, while diclofenac was discontinued.

On day 29, the pony was re-examined. The symptoms had gradually returned, and the owner had restarted treatment with oxytetracyclin/polymyxin B [Terramycin-Polymyxin B (see footnote 2)]. There was a diffuse stromal edema in the ventronasal cornea, surrounding several white “spots” of approximately 1 mm diameter. The left pupil was slightly miotic consistent with mild anterior uveitis, but successfully dilated after 1 drop of 1 % atropine eye drops (Atropin Minims^13^). Treatment with oxytetracyclin/polymyxin B [Terramycin-Polymyxin B (see footnote 2)], idoxuridine eye ointment [Iducher (see footmote 12)] and diclofenac eyedrops [Voltaren Ophtha (see footnote 6)] as described above was restarted. Again, *L. monocytogenes* was cultured from a bacterial swab from the corneal lesions. Around day 45, the horse was judged sound by the owner, and all medication was discontinued.

On day 76, the same symptoms recurred. Treatment with ciprofloxacin eye drops [Cilox (see footnote 9)] QID was started, and maintained for 4 weeks. In a telephone interview 6 years later, the owner reported that the pony had shown no symptoms of ocular disease since this treatment. They had, however, also stopped using haylage in the paddocks.

### Case 3

A 10-year-old Icelandic horse, which was kept in a group and fed plastic wrapped silage, was referred to the NSVS for evaluation of kerato-conjunctivitis in the right eye of 1 month duration. Despite initial improvement after treatment with fucidic acid eye drops [Fucithalmic (see footnote 1)] twice daily for 10 days, the symptoms recurred when treatment was stopped. The same treatment was re-initiated, this time without noticeable effect.

The horse was presented with a mild blepharospasm of the right eye, a moderate periorbital edema with moderately hyperaemic and chemotic palpebral conjunctivae. Slightly nasal to the centre of the cornea, there was a very faint grayish epithelial opacity, only visible with a focal light source. No further pathology was seen. A bacterial swab was collected as previously described. The fluorescein dye test was negative. Treatment with chloramphenicol eye ointment [Chloramphenicol (see footnote 3)] three to four times daily and diclofenac eye drops [Voltaren Ophtha (see footnote 6)] two to three times daily was initiated awaiting sampling results.

At day 6, *L. monocytogenes* was cultured from a bacterial swab. The topical antibiotic treatment was changed to ciprofloxacin eyedrops [Cilox (see footnote 9)] four times daily for 4 weeks. At day 30, the owner reported that the symptoms were reduced, but that a mild epiphora was still present. The treatment was therefore prolonged for another 14 days.

A few days after discontinuation of treatment, the symptoms recurred. At re-examination on day 48, the clinical findings were identical to those described at day 1. A new bacterial swab revealed continuous growth of *L. monocytogenes* without changes to the sensitivity pattern. Due to the poor clinical effect of ciprofloxacin, treatment was changed back to chloramphenicol [Chloramphenicol (see footnote 3)] eyedrops 5 times daily (ointment at night) for 6 weeks. Around day 90, the owner discontinued the treatment without further veterinary consultation. The following days symptoms as originally described recurred. On day 104 the horse was hospitalized at the NSVS.

The diffusely outlined epithelial opacity was still faint, but now covering more than 50 % of the corneal surface. A few superficial stromal, very thin blood vessels were now seen progressing approximately 7 mm from the dorsal limbus. Mild uptake of fluorescein dye was found in a 5 mm diameter area located 5–10 mm from the ventronasal limbus.

The horse was treated with a continuous flow of chloramphenicol eyedrops [Chloramphenicol (see footnote 3)] via a superior subpalpebral lavage system attached to a continuous infusion pump system (see footnote 10) for 14 days and systemic procaine penicillin [Penovet vet (see footnote 7); 20,000 IU i.m.] twice daily for 10 days. At day 118 there were no signs of conjunctivitis, but still a faint epithelial opacity. The horse was sent home on another 14 days of chloramphenicol eye ointment [Chloramphenicol (see footnote 3)] four times daily.

According to a telephone interview with the owner at day 191 the horse had not showed signs of ocular disease after termination of treatment at day 132. Around day 280, the horse was presented to a field practitioner with bilateral conjunctivitis. Bacterial swabs were collected from both eyes and submitted to the NSVS for culturing. A mixed flora was cultured, dominated by *Staphylococcus aureus* in the right eye and *Pasturella sp* in the left eye; both sensitive to chloramphenicol. *L. monocytogenes* was not found at this stage.

### Case 4

An 8-year-old Icelandic pony with a six month history of unilateral recurrent kerato-conjunctivitis, affecting the left eye was presented to the horse clinic at Bjerke Equine Hospital, Oslo. The owner had first noticed a problem approximately 5 months earlier whilst the horse was at pasture and described symptoms of recurrent blepharospasm with ocular discharge. The eye was treated topically on several occasions with no lasting improvement, however the examination findings and medications used are not known. Three months after the symptoms were first noticed, a superficial corneal ulcer associated with a corneal foreign body was observed by the attending veterinary surgeon. During the past 2 months, the horse had been fed plastic wrapped haylage from the ground. The foreign body was removed and the loose corneal epithelium surrounding the ulcer was debrided. The eye was medicated with topical chloramphenicol eye ointment [Chloramphenicol (see footnote 3)] three times daily for 1 week.

Two months after removal of the foreign body the horse was presented to Bjerke Equine Hospital due to persistent problems with the eye. On examination the eye showed moderate blepharospasm with slight epiphora. No sign of anterior uveitis was evident. The cornea had a dull appearance and there was a small area of epithelium, approximately 1 cm in diameter, in the central cornea which had an uneven, stippled appearance. Fluorescein and Rose Bengal staining was negative. No further abnormalities were found.

The cornea was treated topically with neomycin sulphate, polymyxin B sulphate and dexamethasone [Maxitrol (see footnote 9)] three times daily. The owner noticed an immediate improvement and on re-examination 1 week later the horse exhibited no blepharospasm or ocular discharge. The corneal surface was shiny and smooth. The only abnormality noticed was several very small, <1 mm, white sub-epithelial opacities in the central cornea. Medication was continued for one week.

Two to three days after treatment was discontinued, the owner noticed that the horse was showing signs of ocular discomfort once more. Treatment with topical 0.1 % dexamethasone disodium phosphate (Spersadex^14^) three times daily was instigated. The eye showed no improvement and was presented for re-examination 2 weeks later.

At re-examination the horse showed obvious blepharospasm and significant epiphora. The ventral palpebral conjunctiva was chemotic. A superficial corneal ulcer was present in the central corneal area, approximately 8 × 5 mm, which was associated with a very mild, diffuse edema. The pupil was moderately miotic, indicating mild anterior uveitis. No corneal neovascularization was evident. Fluorescein testing resulted in a faint staining of the ulcer margins.

The epithelial surface was carefully scraped and slides prepared for cytology, thereby removing loosely adherent epithelium in an area of approximately 15 × 15 mm. A swab for bacterial and fungal culture was collected from the affected area of the cornea.

**Fig. 1:**
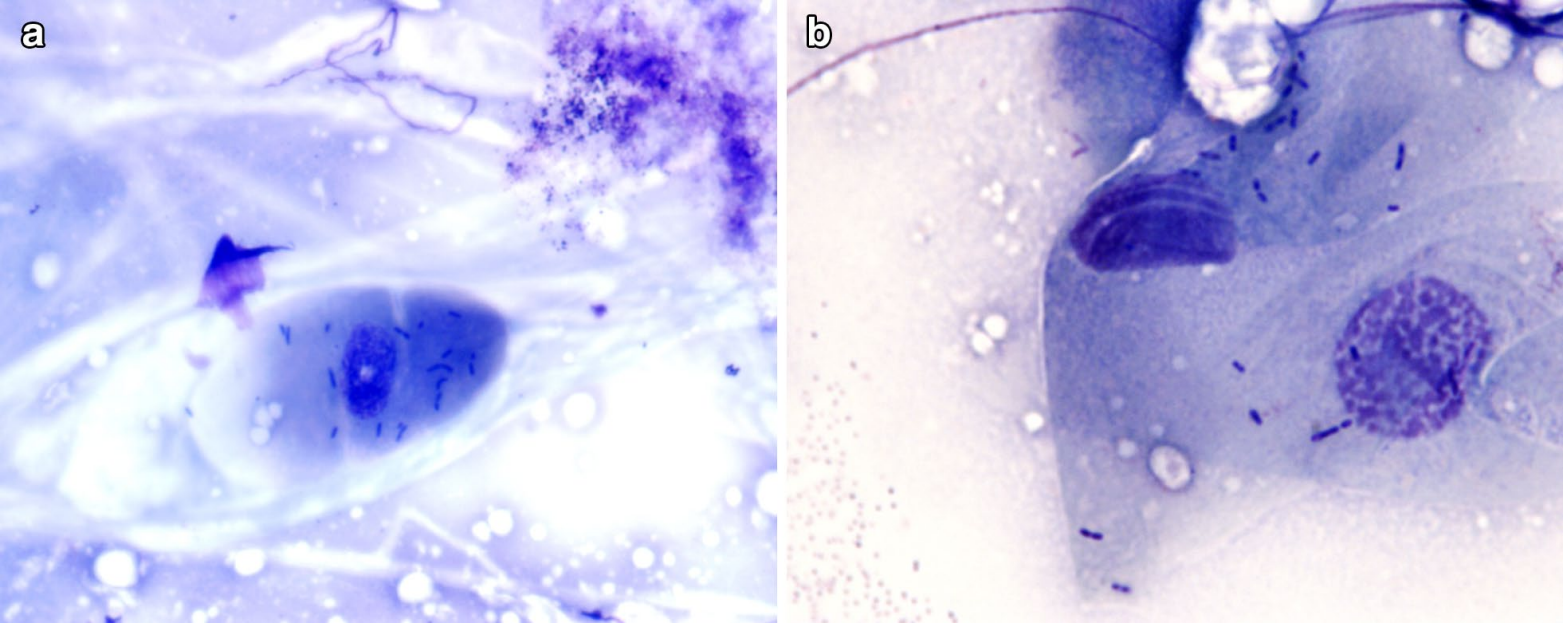
**a**. Squamous epithelial cell with typical rod-shaped bacteria on the surface. Note the absence of inflammatory cells. ×600 Modified Wright stain. **b** The rod-shaped bacteria are suggestive of *L. monocytogenes*. ×1000 Modified Wright stain

Whilst awaiting the results for the samples taken, the horse was hospitalized and the eye treated topically with oxytetracyclin/polymyxin B [Terramycin-Polymyxin B (see footnote 2)] three times daily. A 1 % atropine eyedrop [Atropin Minims (see footnote 13)] was applied on one occasion which resulted in a profound mydriasis. In addition the horse was treated with systemic flunixin meglumine (Finadyne Vet^15^; 1,1 mg/kg iv) once daily for a period of 5 days. After 5 days the horse showed no ocular discomfort or inflammation; the ulcer had healed and fluorescein staining was negative. At this point the horse was discharged and the owner continued with the antibiotic treatment for a further 7 days. Cytology revealed the presence of many rod-shaped bacteria in short chains indicative of *L. monocytogenes*. The bacteria were associated with squamous epithelial cells (Fig. 1). From the corneal swab it was cultured a sparse, mixed bacterial growth dominated by *L. monocytogenes*.

The horse was re-examined 5 days after cessation of antibiotic treatment, 12 days after discharge from the hospital. On examination the horse showed no blepharospasm or ocular discharge. The conjunctivae were not inflamed and the cornea was smooth with a healthy, shiny corneal surface. No corneal ulcer, edema or neovascularisation was evident. Fluorescein and Rose Bengal staining was negative. Three small, <1 mm, white, sub-epithelial opacities in the area of previous ulceration were still visible with magnification. No bacterial growth was cultured from a corneal swab. Four months after discharge, the owner reported no sign of recurrence.

A brief overview of key clinical observations, culturing results and treatments in all four cases is given in Table 1.

**Table 1:** Key clinical observations, culturing results, topical and systemic treatments in four cases of equine keratitis

Case No	Day after initial examination	Clinical diagnoses	Bacterial growth after culturing^a^	Topical medication (frequency)^b^	Systemic medication (days)
1	1	Conjunctivitis, moderate central superficial keratitis^1^	*Micrococcus* spp.—moderate *L. monocytogenes*—sparse	*Chloramphenicol (TID* – *QID)*	–
	15	Unchanged^1^	*L. monocytogenes*—sparse	*Ampicillin, Gentamicin (QID)* *Diclofenac (QID)*	*Procaine penicillin, Gentamicin (5* *days)*
	55	No symptoms^2^		–	–
	69	Conjunctivitis, moderate central superficial keratitis^1^	Normal flora dominated by *Micrococcus* spp. and *L. monocytogenes*	*Ciprofloxacin (QID)* *Diclofenac (QID)*	–
	114	Unchanged^1^	*L. monocytogenes* abundant before scraping/very sparse after	Penicillin-Na, Gentamicin (CF)	Procaine penicillin (10 days) Vedaprofen (X days)
	128	Central superficial keratitis improved^1^	No listerial growth (selective enrichment)	Gentamicin (CF)	–
	140	Conjunctivitis, moderate central superficial keratitis^1^		Penicillin-Na, Gentamicin (CF)	–
	150 (*released against medical advice)*	central superficial keratitis improved^1^		*Oxytetracyclin* - *PolymyxinB (TID)*	–
	178 (*euthanized—unrelated reason)*	Unchanged^2^			
2	1	Conjunctivitis, mild–moderate central superficial keratitis^1^	*L. monocytogenes*—sparse	*Oxytetracyclin* - *PolymyxinB (QID* *), Idoxuridine (QID), Diclofenac (TID)*	–
	12	Mild keratitis^1^		*Oxytetracyclin* - *PolymyxinB (QID 7* *days),* *Idoxuridine (QID–7* *days)*	–
	29	Conjunctivitis, mild–moderate central superficial keratitis, mild anterior uveitis^1^	*L. monocytogenes*—moderate (pure culture)	*Oxytetracyclin* - *PolymyxinB (QID)*, *Atropin (1 single drop), Idoxuridine QID, Diclofenac TID*	–
	45	No symptoms^2^		–	–
	76	Conjunctivitis, mild–moderate central superficial keratitis^1^		*Ciprofloxacin (QID* 28 days *)*	–
	2200	No further symptoms^2^			–
3	1	Conjunctivitis, mild central superficial keratitis^1^	*L. monocytogenes*—moderate (pure culture)	*Ciprofloxacin (QID)* *Diclofenac (BID*-*TID)*	–
	30	Conjunctivitis^2^		*Ciprofloxacin (QID)*	–
	44	None^2^		–	–
	48	Conjunctivitis, mild central superficial keratitis^1^	*L. monocytogenes*—sparse (pure culture)	*Chloramphenicol (Eyedrops 5x/day* *Ointment at night 42* *days)*	–
	90	None^2^			
	104	Conjunctivitis, mild–moderate central superficial keratitis^1^	*L. monocytogenes*—very sparse (pure culture)	Chloramphenicol (CF)	Procaine penicillin (10 days)
	118	Mild keratitis^1^		*Chloramphenicol (QID* – *ointment 14* *days)*	–
	162	No symptoms^2^			
4	1	Mild central superficial keratitis^1^		*Neomycin* - *Polymyxin B*-*Dexamethasone (TID 14* *days)*	–
	16	Conjunctivitis^2^		*Dexamethasone (TID)*	–
	28	Conjunctivitis, mild–moderate central superficial keratitis (ulcerative), mild anterior uveitis^1^	Sparse, mixed bacterial growth dominated by *L. monocytogenes*	Oxytetracyclin-PolymyxinB (TID 5 days) Atropin (1 single drop) *Oxytetracyclin* - *PolymyxinB (TID 7* *days)*	Flunixin (5 days)
	45	None^1^	No bacterial growth		
	160	No symptoms^2^			

Treatment prior to referral and short term medications awaiting culture results not included in the table. ^1^ Veterinary diagnoses, ^2^ Owner observation

*italic treatment outside clinic*, *BID* treatment twice daily, *TID* treatment 3 times daily, *QID* treatment 4 times daily, *CF* continuous flow via subpalpebral lavage system, underlined: antibiotic treatments

^a^Typically 3–5 days after sampling

^b^Topical antibiotics initiated after received culture report

### Bacteriological examination and findings

In all cases, samples for microbiology were collected during the initial examination and for follow-up and evaluation of treatment response. Sterile cotton swabs in Amies transport medium without charcoal (Copan M40 Transystem^16^) were used for transporting swab specimens to the bacteriological and mycological diagnostic laboratory at the NSVS, where culturing was started within a few hours after sampling.

### Media and cultivation

The samples were in all cases incubated on parallel agar plates (blood agar base no 1)^17^ with 5 % cattle blood of which one was incubated at 37 °C in 5 % CO_2_ atmosphere for 24 h while the other was incubated anaerobically for 48 h. The samples were also inoculated in serum broth (see footnote 17) and incubated at 37 °C in 5 % CO_2_ atmosphere for 24 h for enrichment of growth for secondary plating on blood agar in case of no visual growth on primary blood agar after 24 h.

In addition, the samples were routinely incubated on bromothymol blue agar used for detection of bacteria in the *Enterobacteriaceae* family and other Gram-negative rods.

Materials from samples were also directly Gram stained to detect any bacteria in the sample before cultivation.

### Culture examination and biochemical tests

Growth on the plates was examined followed by recording of morphological and other features of the colonies such as occurrence of any haemolytic zones. Gram stained colony material was examined and cell morphology recorded. Potential *Listeria*-like colonies were secondarily streaked on blood agar plates at right angles close to simultaneously streaked cultures of *Staphylococcus pseudintermedius* and *Rhodococcus equi* to detect any CAMP phenomenon.

Biochemical tests of *Listeria*-like colonies included the catalase test, fermentation of glucose, degradation of aesculin, hippurat and H_2_S. The methyl red, Voges Proskauer and motility tests (in semi-liquid agar grown at 22 and 37 °C) were also performed.

Bacteriological findings from all eye swab samples collected during the course of the disease in all cases are summarized in Table 1. Colonies suspected to be *L. monocytogenes* were 1.0 mm in diameter, convex and grey to white in colour with a clear zone of β-haemolysis extending barely (about 0.5 mm) outside the colony edge. There was no growth on bromothymol blue agar from any of the corneal swabs. There was no evidence of any antibacterial agent in the material from initial corneal swabs as seen from the control growth of *S. pseudintermedius* on the blood agar incubated in CO_2_ atmosphere. The same type of colony was detected on blood agar after cultivation from the LB enrichment broth after one, two and 3 days. In all four cases culturing revealed a low to moderate number of colonies in pure culture with similar cultural characteristics to *L. monocytogenes* (Table 1).

### Phenotypical and biochemical features of β-hemolytic colonies

The β-hemolytic colonies from the corneal swabs contained bacteria that were Gram positive, short coccoid and regular sized rods. The isolates were catalase positive, fermented glucose, did not metabolise aesculin or hippurate, produced H_2_S, and were negative on methyl red and Voges Proskauer tests. They were non-motile on semi-solid agar at 37 °C, but motile at 22 °C. They showed weak CAMP reaction with β-toxic *S. pseudintermedius* but were CAMP negative with *R. equi.* The biochemical profile and phenotypic features suggest that the colonies were *L. monocytogenes*.

### Serotyping and antimicrobial susceptibility testing

Incubation of the test samples and cultures of *L. monocytogenes* reference strains CCUG 15527 (serotype 1) and CCUG 7995 (serotype 4) for 24 h at 37 °C demonstrated that the isolated *L. monocytogenes* of all cases belonged to the O serotype 1 judged by the phenotypical similarity of the colonies and their haemolytic zones.

Agglutination tests performed on glass slides with antisera against *Listeria* O type 1 (see footnote 17) and *Listeria* O type 4 (see footnote 17) also showed the same result. Antimicrobial susceptibility tests were carried out using the tablet diffusion method with twelve different antibacterial drugs including penicillin, ampicillin, cefalexin, amoxycillin, amoxycillin with clavulanic acid, sulphadiazine/trimethoprim, tetracycline, gentamicin, lincomycin, chloramphenicol, fucidic acid, enrofloxacin, and colicin. Agar diffusion on Müller-Hinton (MH) agar (see footnote 17) was used with tablets containing antibiotics (Neo-Sensitabs)^18^ for the susceptibility tests. The tests showed that isolates of *L. monocytogenes* from all four cases were found to be either moderately susceptible or in some cases resistant towards fucidic acid. The isolates of *L. monocytogenes* from case 3 and 4 (both from the initial and follow-up isolations) were found to be intermediate susceptible towards enrofloxacin. The isolates were found to be susceptible to all other antimicrobial drugs tested.

### General discussion

The four cases of kerato-conjunctivitis in horses associated with *L. monocytogenes* reported in this study represent the first reported cases in Scandinavia.

*L. monocytogenes* is a facultative intracellular pathogen widely distributed in the environment where most animals are exposed to the organism during their lifetime. The bacterium causes disease either when the host’s immune system is compromised or when the bacterium occurs in numbers high enough to overwhelm the host’s defence mechanisms [2, 9, 11]. Although the common route of infection is oral, the bacterium may also enter the body through the nasal mucosa or open wounds. The conjunctiva has also been mentioned as a possible entrance [24, 34]. In contrast to the high frequency of ocular signs of listeriosis described in cattle [2], ocular listerial infections seem to be rare in horses with only two cases reported so far [25, 26].

All cases reported here had symptoms of corneal disease for an extended time period prior to referral. Hence, it is not possible to determine the pathogenesis in these cases with certainty. *L. monocytogenes* serotype O type 1, a common strain found in the environment, in silage and in systemically infected animals [22], was cultured from all cases reported here. This is in agreement with the theory of silage as the most likely source of infection, and with the findings of Evans et al. [26] that ocular listerial infections seem to be caused by common strains of the bacterium that are also found in systemic infection.

The intact and healthy cornea is highly resistant to bacterial infection, due to a continuous flow of a tear film with antimicrobial properties, as well as the physical barrier of a healthy epithelium [27]. It has, however, been shown that *L. monocytogenes* may penetrate into undamaged corneal epithelial cells of guinea pigs [28]. Further, conjunctivitis and/or kerato-conjunctivitis has been induced experimentally in guinea pigs and rabbits simply by direct inoculation of *L. monocytogenes*, while similar direct inoculation of a variety of other bacteria did not cause infection [29]. In fact, this characteristic ability to cause conjunctivitis and keratitis was in 1939 even proposed as a method to identify an infectious culture as *L. monocytogenes* [30]. It has also been shown that the infectivity of listerial cultures after direct inoculation in rabbits is dose dependent [9]. Whether direct inoculation would cause kerato-conjunctivitis in a horse remains speculative, not least given the more than 3 times thicker corneal epithelium in horses [31, 32] compared to rabbits [33].

There is a well-known general association between silage feeding and listeriosis [5, 34–37]. In horses, systemic *L. monocytogenes* infections have been associated with silage feeding [22, 26], feeding on poor quality hay [17] or grazing on spray and flood-irrigated grass clover pasture [14]. Plastic wrapped forage (silage or haylage) is commonly used to feed horses in Norway, especially during the winter season, and appears to be the most likely source of *L. monocytogenes* in the cases described here. Grass silage produced in cool, wet climates tends to have lower sugar levels and higher moisture contents than silage produced in warmer climates. This may result in a poorer and slower fermentation, which provides better conditions for the proliferation of *L. monocytogenes* in the silage [38]. Poor quality hay or soil-contaminated forage represent other possible sources of infection in these cases.

In several studies, feeding of animals with silage placed at or above head height in such a way that silage material could fall into the eyes of the animals has been incriminated as a possible predisposing factor of listerial eye infection [35, 37, 39]. Such feeding routines are not common in horses. Still, frozen forage or forage made into a hard bale as in silage or haylage may be difficult to pretend, and horses may pull forage out of the bale and throw it up in the air, increasing the likelihood of ocular exposure to contaminated material. Hence, it appears possible that feeding routines, although not further investigated, may have played a role in these cases.

Corticosteroid treatment is known to increase the infectivity of *L. monocytogenes* in laboratory animals [1]. The case of equine listerial keratitis reported by Sanchez et al. [25] had been treated with topical steroids prior to referral. Only one of the horses included in this report (case 4) had been treated with topical steroids prior to the identification of *L. monocytogenes,* and no other signs of immunosuppression were identified in any of these cases.

*L. monocytogenes* is often detected in low numbers by cultivation, sometimes together with other bacteria. This observation is important for the bacteriologist when cultivating samples from cases of keratitis, and may be related to the intracellular growth of *L. monocytogenes.* The low number of *Listeria* bacteria seen on culturing may have contributed to the low number of reported cases in the literature. Also, as noted by other authors there may be lacking awareness among clinicians as well as bacteriologists regarding the potential role of *L. monocytogenes* in ocular infections [9, 11, 26]. A thorough cytologic evaluation of corneal scrapings was found to be of great help in these cases, but comprehensive sampling is crucial as indicated by positive findings in only one out of several slides at the last recurrence in case 1.

Listerial infections are commonly treated with penicillins or tetracylines, which may be effective in early stages of systemic disease. However, if severe symptoms have developed prior to initiation of intensive antibiotic treatment, the outcome is commonly fatal [2].

Combined use of gentamycin and penicillin was found to be more effective than either of the antibiotics used alone in treatment of experimental listerial keratitis in rabbits [9]. The same combination of antibiotics was used systemically as well as topically for successful treatment of a severe case of human listerial keratitis [9].

Based on these findings, penicillins (ampicillin or penicillin G) and gentamicin were used systemically and topically in case 1. Some comments need to be made regarding this combined treatment: The low lipophilicity of Penicillin G likely restricts its ability to penetrate the barrier of the lipophilic corneal epithelium. Although topical penicillin treatment has been recommended for use in humans with ocular surface disease [40], the poor penetration of the epithelium may well limit the efficacy of the drug against intracellularly located strains of *Listeria.* Notable in this context is the fact that the horses in this study on many occasions had intact epithelium covering the corneal lesions, as demonstrated by negative Fluorescein dye tests. Further, aminoglycosids may be inactivated in plasma in approximately 8–48 h in the presence of ß-lactams [41]. In order to minimize inactivation, a fresh mixture for continuous flow treatment was prepared daily in case 1. The extent of potential aminoglycoside inactivation was not investigated. Despite these factors, this combined treatment appeared to be effective, but was probably stopped too early.

Various other recommendations have been published with regards to treatment of ocular listerial infections. Both the previously reported cases of listerial kerato-conjunctivitis in horses were treated successfully with kinolones (alone or in combination) [25, 26]. Based on these reports, the kinolone Ciprofloxacin was used in cases 1–3, but this treatment was only successful in case 2. These treatments were carried out by the horse owners, and it is possible that the lack of success may have been related to owners’ compliance. On the other hand, failure of listerial keratitis to respond to treatment with the kinolone Ofloxacin despite in vitro susceptibility has been reported in a human patient [10]. Interestingly, the isolates from cases 3 and 4 were found to be intermediate susceptible towards enrofloxacin, which was the only kinolone available for susceptibility testing.

The isolates described here were found to be susceptible to most of the antibiotics tested, but intermediately susceptible or in some cases resistant to fucidic acid. Fucidic acid is commonly used as a “first choice” antibiotic to treat suspected bacterial keratitis or conjunctivitis in Norway, and had been used prior to referral at least in cases 1 and 3.

Despite extremely prolonged unsuccessful treatment with chloramphenicol in case 3, hospitalization and intensified treatment with continuous flush using the same antibiotic led to final resolution of the symptoms, although this final treatment was supplemented by systemic penicillin for 10 days. Although the possibility of re-infections cannot be completely ruled out, we believe recurrences of symptoms in these horses after termination of treatment were related to lack of successful elimination of the infective agent, not least due to the consistent unilaterality of the clinical signs. In summary, we believe that several antibiotics may be used successfully for treatment, but that extra attention must be paid to intensity and duration of treatment once *L. monocytogenes* has been identified in cases of equine kerato-conjunctivitis. Cases should also be monitored closely after termination of antibiotic treatment. Difficulties in treatment similar to those described here have also been reported in human ophthalmology [9–11, 42].

## Conclusions

*L. monocytogenes* should be considered a potential pathogen in cases of indolent bacterial kerato-conjunctivitis in horses, especially in animals fed with silage or haylage.

Special attention may be required from clinicians as well as bacteriologists and clinical pathologists in order to identify the bacteria. The generally poor response to treatment and frequent recurrences in the cases described here indicate that clinicians should pay extra attention to the choice of antimicrobial drugs, and to the intensity and duration of the treatment regimen if *L. monocytogenes* is identified from equine keratitic lesions. Further research is needed to determine whether *L. monocytogenes* may act as a primary pathogen, or rather as a secondary invader in cases of pre-existing corneal lesions.

## Footnotes

1 Dechra Veterinary Products A/S, Uldum, Denmark.

2 Pfizer Inc, New York, USA.

3 Nycomed Pharma A/S, Asker, Norway.

4 Bristol-Myers Squibb Company, New York, USA.

5 Schering-Plough International, Kenilworth, New Jersey, USA.

6 Novartis Healthcare A/S, København, Denmark.

7 Boehringer Ingelheim Danmark A/S, København, Denmark.

8 Kela N.V., Hoogstraten, Belgium.

9 Alcon Laboratories, Inc., Fort Worth Texas, USA.

10 Mila International, http://milainternational.com/.

11 Actavis Group, Hafnarfjordur, Iceland.

12 Farmigea S.p.A., Pisa, Italy.

13 Bausch & Lomb U.K. Ltd., Surrey, England.

14 OmniVison AB, Danderyd, Sweden.

15 Intervet International B.V, Boxmeer, The Netherlands.

16 Copan Italia S.p.A., Brescia, Italy.

17 Difco Laboratories Inc., Detroit, Michigan, USA.

18 Rosco, Taastrup, Denmark.
